# BMR-YOLO: A deep learning approach for fall detection in complex environments

**DOI:** 10.1371/journal.pone.0335992

**Published:** 2025-11-07

**Authors:** Hang Ren, Ping Lan

**Affiliations:** College of Information Science and Technology, Xizang University, Lhasa, China; Kafkas University: Kafkas Universitesi, TÜRKIYE

## Abstract

Traditional fall detection methods face significant limitations in complex environments, particularly under occlusion and poor lighting conditions. To address these challenges and enhance the detection accuracy in intelligent real-time monitoring systems, this study proposes an optimized BMR-YOLO framework based on the YOLOv8n architecture. Our approach introduces four strategic improvements to effectively overcome environmental constraints. First, we enhance the backbone network by incorporating a BiFormer vision transformer with dual-layer routing attention, enabling dynamic allocation of computational resources, while improving both computational efficiency and feature extraction performance. Next, we replace the C2f module in the backbone with C2f_rvb, enhancing the model’s ability to handle multi-scale features while reducing computational requirements. Furthermore, we strengthen the detection head by adding the MultiSEAM attention mechanism, significantly improving the detection accuracy of occluded objects. Finally, we optimize the regression process by replacing the traditional CIoU loss with a direction-aware SIoU metric, thereby improving both the localization accuracy and training stability of bounding box regression. To validate our method, we constructed a comprehensive dataset, BMR-fall, containing over 10,000 annotated images that capture various fall scenarios, and performed cross-validation using the UR fall detection dataset. Experimental results demonstrate that BMR-YOLO achieves a notable improvement in mAP@0.5, rising from 0.852 to 0.899 on our proprietary dataset, while maintaining a low computational cost of 6.5 GFLOPs. Comparative analysis with existing methods shows that BMR-YOLO outperforms them under occlusion and lighting variation conditions, confirming the model’s robustness and practical applicability for real-world deployment.

## 1 Introduction

Fall detection plays a crucial role in ensuring personal safety. Falls not only jeopardize athletes’ careers but also disrupt the daily lives of younger individuals, while posing particularly severe risks to the elderly. With the aging population growing rapidly, the likelihood of falls among older adults is increasing, often resulting in serious physical injuries. Consequently, the demand for efficient and accurate fall detection systems have become more urgent. By enabling timely identification and intervention, these systems can effectively mitigate fall risks, provide essential assistance to the elderly, and ultimately enhance their safety and quality of life. Currently, fall detection primarily employs object detection methods from deep learning. These approaches are classified into two types: the first type consists of two-stage systems such as R-CNN detection methods. This includes R-CNN [1], Faster R-CNN [2], and Mask R-CNN [3]; while the second category features single-stage algorithms like SSD [4], RetinaNet [5], and the YOLO [6] family. To address the sudden nature of fall events, Hazlina et al. [7] proposed a system that analyzes video frames using object detection algorithms to identify falls in real time and send alerts via email. Wang et al. [8] modified the loss function and incorporated an attention mechanism into the core network to enable real-time identification of human falls. Raza et al. [9] utilized YOLO and its variants to detect falls and various actions of multiple people in the same scene. Yangsenchen et al. [10] introduced a pose estimation-based method and an auxiliary detection method based on YOLOv5; the improved algorithm effectively detects falls or daily activities in each frame of the image and provides real-time feedback. Dara ROS et al. [11] proposed a motion-based multi-camera fall detection system that is implemented by detecting human bodies and tracking their movement in real time. This framework is adaptable to various use cases, this makes it possible to merge various object detection approaches and aids in analyzing video from different angles. Guanghuichen et al. [12] introduced a novel convolutional network module, it is designed with novel features aimed at improving the network’s performance and efficiency, the Higher-Order Coordinated Attention Module, and combined it with a lightweight convolutional module to optimize and optimize the YOLOv5s model to enhance its performance and achieve better efficiency. By integrating this approach, the model is able to manage complex tasks with greater efficiency, resulting in enhanced object detection accuracy and faster processing speeds. This resulted in the development of an innovative fall detection model, YOLOv5s-CAGn-GSConv. This method improves detection accuracy significantly and reduces the model’s parameter requirements in an efficient manner, there by optimizing computational efficiency while maintaining performance. Challenges, including variations in object scale and occlusion of standard objects, pose significant difficulties, detection accuracy can be low. To tackle this, Xiaolin Li et al. [13]. Proposed a drone-based algorithm for detecting miniature objects based on the improved YOLOv8n model incorporating a new hybrid module that combines the C2f structure in YOLOv8n with the Efficient Attention mechanism, introducing SPD-Conv to replace all downsampling modules, and implementing a dynamic head design (dyHead) while using Soft-NMS instead of traditional non-maximum suppression in the image post-processing phase to reduce missed detections caused by density and occlusion issues. To accurately detect occluded objects, Zhe Lin et al. [14] extended the YOLOv5 architecture by initially introducing a coordinate attention module in the feature extraction layer, then employing Complete-IOU (CIoU) to replace the original loss function, and finally proposing kCIoU-NMS to overcome the shortcomings of IoU. The results of the experiment show that the suggested approach delivers exceptional effectiveness and considerable robustness in detecting occluded objects. Existing fall detection algorithms primarily focus on improving accuracy in simple scenarios, frequently overlooking the effects of challenging environments, such as low lighting and crowd obstruction. In conditions with lighting changes and partial body occlusion, the performance stability and accuracy of existing algorithms still have significant room for improvement. To resolve this challenge, the research proposes BMR-YOLO, an algorithm specifically designed for fall detection, designed to improve detection performance in challenging environments, particularly those with partial occlusion and low lighting conditions. The main contributions of this research include:

1. At the end of the backbone, the BiFormer [15] attention mechanism has been incorporated to enhance the representation of target regions within the feature map. This mechanism facilitates bidirectional information transfer, which improves the model’s ability to distinguish key areas of interest by effectively reducing the impact of background noise. As a result, it substantially improves the model’s capacity to identify key behavioral patterns while also lowering the rate of false positives.
2. To enhance the model’s ability to extract multi-scale features and reduce the computational overhead, we propose introducing the C2F_RVB module into the network backbone as a replacement for the traditional C2F module. This substitution enables the model to process multi-scale information more efficiently while maintaining lower computational complexity, thereby improving both performance and efficiency.
3. The MultiSEAM [16] attention mechanism has been incorporated to enhance the detection head, effectively addressing object occlusion while enhancing the model’s precision in detecting partial obstructions of the human body.
4. To address the instability in target box regression, we improved the loss function in YOLOv8n by replacing CIoU [17] with SIoU [18], which resolves issues such as divergence during training and further enhances accuracy.

## 2 Related work

### 2.1 Fall detection in obstructed environments

Public safety incidents involving falls due to crowding or stampedes have been increasing. While minor falls may cause limited harm, severe falls can be life-threatening. Effective fall detection is essential for timely intervention and risk mitigation. Traditional detection methods perform well in controlled settings but often suffer from low accuracy, high false-positive rates, and high computational costs in complex environments. With advances in deep learning, fall detection in public spaces has seen improvements in both efficiency and accuracy. Our proposed BMR-YOLO addresses these issues by reducing computational load, improving accuracy, and minimizing missed detections, thereby enhancing public safety and personal security.

### 2.2 Attention module

SENet [19] significantly improves the representational capacity of neural networks by introducing an adaptive channel weighting mechanism. SENet primarily involves two operations: Squeeze and Excitation. Applied global pooling to average all channels in order to obtain a reasonable result is the function of the Squeeze operation, while the excitation operation determines the weights of the channels through the use of two fully connected layers, employing a sigmoid activation function to calculate the channel-specific weight parameters. The initial feature map is multiplied by the weights derived from all channels in the final step. This operation adjusts the contribution of each channel, enhancing relevant features and reducing noise, applying weighted attention to emphasize the key channels’ information. Woo et al. [20] incorporated channel and spatial attention modules together, introducing CBAM to enhance the representational capability of CNNs. To reduce the loss of information and strengthen global dimensional interactions, Liu et al. [21] made adjustments to CBAM and introduced GAM, which improved accuracy but often resulted in increased model complexity and greater computational demands. To address this, Liezhu and his colleagues proposed a new method called the BRA attention mechanism, integrating it into the Vision Transformer to create BiFormer. This approach uses a two-layer routing method to provide more flexible computational allocation and better content awareness. This dual-layer routing reduces computational overhead while maintaining model performance.

## 3 Methods

### 3.1 YOLOv8 model

The YOLOv8 architecture builds upon the ideas of the YOLOv5 structure, it enhances object detection, image classification, and instance segmentation performance based on YOLOv5. YOLOv8 includes five different models, with YOLOv8n having the lowest computational complexity and parameter count. YOLOv8 introduces extensive modifications and optimizations to its configuration files. These enhancements enable YOLOv8 to achieve higher accuracy and faster speeds in object detection compared to YOLOv5, YOLOv6 [22], and YOLOv7 [23]. In the YOLOv8 backbone, the C3 module from YOLOv5 is replaced with C2F, contributing to a more lightweight design. It retains the CSPNet concept from YOLOv5 and the SPPF module in its architecture. The detection head features a Decoupled-Head, this can improve the performance of both the training and inference stages. YOLOv8 adopts an Anchor-Free method in the prediction head, enabling direct prediction of object positions and sizes without relying on anchor boxes, thus eliminating the need for generation and filtering. For the loss function, different improved loss functions are used for different classifications and optimization of regression losses.

### 3.2 BMR-YOLO

**Fig 1:**
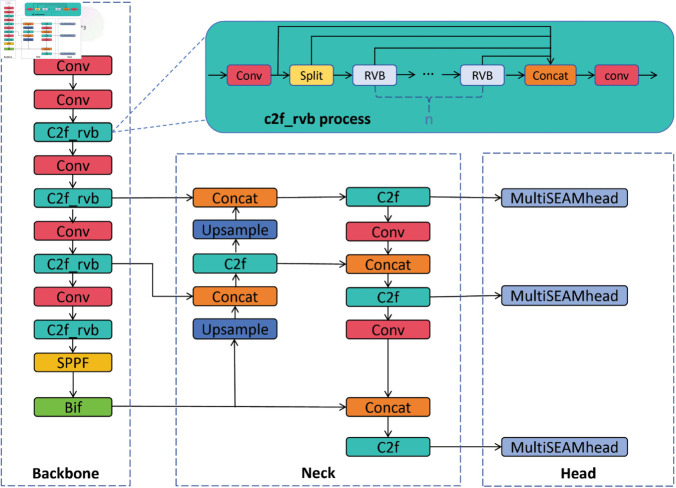
BMR-YOLO model architecture. The proposed framework enhances YOLOv8n by integrating four key modules: the BiFormer attention for global feature awareness, C2f_rvb for multi-scale feature extraction, MultiSEAM attention in the detection head for occlusion robustness, and the SIoU loss for improved box regression. This design specifically targets challenges in complex fall detection scenarios, such as occlusion and low-light conditions.

Fig 1 presents the BMR-YOLO structure diagram. By enhancing the network architecture of YOLOv8n, we achieved our experimental goals. Through multiple experiments, we introduced the BiFormer (BiF) attention mechanism at various positions within the Backbone layer. Based on experimental findings, we determined that adding the BiF attention module at the end of the Backbone allows dynamic sparse attention, helping the network achieve more flexible computation allocation and content awareness of global information. Additionally, within the Backbone layer, we replaced the C2f with C2f_RVB[30] to achieve multi-scale feature extraction, reducing the computational power required for real-time detection. Our tests showed that replacing only the C2f_RVB with C2f in the Backbone layer yielded the best results. Furthermore, we integrated the MultiSEAM attention mechanism into the detection head to improve detection accuracy, especially in complex environments. Lastly, to boost detection speed and enable faster convergence, we incorporated the SIoU loss function. The following sections will systematically cover the relevant modules and improvements.

#### 3.2.1 BiFormer attention.

**Fig 2:**
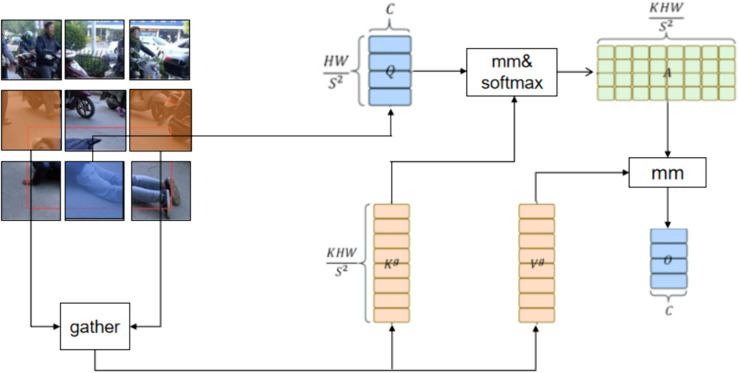
Structure of the BiFormer attention mechanism. This module efficiently models long-range dependencies via a dual-layer routing process. It partitions features into regions, computes affinities, and applies top-k selection to route computation only to the most relevant regions, enabling dynamic sparse attention and reducing computational overhead.

Drawing inspiration from the human visual recognition system, attention mechanisms have become widely used in computer vision to enhance models’ ability to extract spatial features. As a powerful tool, attention mechanisms are capable of capturing long-range dependencies. However, traditional attention mechanisms suffer from two major issues: high memory consumption and significant computational cost. To address these problems, Liezhu et al. introduced a dynamic sparse attention mechanism called “dual-layer routing attention” and integrated it with Vision Transformers, resulting in BiFormer. This dual-layer routing mechanism enables BiFormer to allocate computational resources more efficiently and improves content awareness, thereby boosting object detection performance. The attention structure of BiFormer is illustrated in Fig 2, and its attention mechanism follows three key steps.

(1) Given an input feature map $X\in R^{H*W*C}$, begin by dividing it into S*S non-overlapping sub-regions, ensuring that every region is assigned a distinct feature vector $\frac{HW}{S^{2}}$. Then convert *X* to $X^{\gamma }\in R^{S^{2}*\frac{HW}{S^{2}}*C}$ for subsequent mapping of $Q,K,V\in R^{H*W*C}$. Among them, they are represented by the following three equations: (1) $Q=X^{\gamma }W^{q}$; (2) $k=X^{\gamma }W^{k}$; (3) $V=X^{\gamma }W^{v}$;

(2) Then calculate the eigenvalues of *Q* and *K* separately, multiply the attention matrices of two regions to obtain the affinity adjacency matrix between them, in which mm represents matrix multiplication and softmax represents the implementation of the activation function of the weighted operation. The attention matrix can be represented by this formula: $A^{\gamma }=Q^{\gamma }(k^{\gamma }{)}^{⊤}$.

(3) Finally, remove the tokens with the lowest correlation in matrix $A^{\gamma }$ from the coarse particles. Selectively retain the top k regions with the highest correlation scores in the $A^{\gamma }$ matrix, and then generate a routing index matrix represented as $I^{\gamma }$. This process can be represented by this formula: $I^{\gamma }=topkIndex(A^{\gamma })$.

Therefore, the BiFormer module effectively models long-range dependencies, preserves positional information, and overcomes challenges such as high memory usage and computational cost. We introduce the BiFormer module into YOLOv8’s backbone, enhancing the ability of the backbone network to extract spatial features.

#### 3.2.2 RepViT block module.

**Fig 3:**
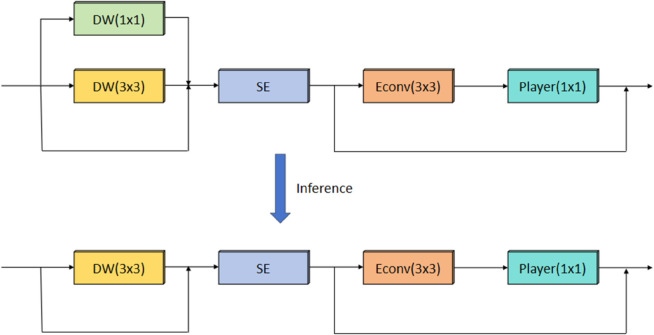
Workflow of the RepViT Block (RVB). The RVB module replaces the standard bottleneck to enhance feature extraction. It combines a main path (with Depthwise Convolution and an SE module) and an interaction path (with 1 × 1 EConv and Projection Layers), synergizing convolutional operations with attention for richer multi-scale features.

The original bottleneck block has limited capability in extracting multi-scale features, while incorporating multi-head attention mechanisms significantly increases the model’s computational cost. The RVB module’s introduction integrates sophisticated feature extraction methods with a refined attention mechanism, improving the model’s ability to focus on essential features and greatly enhancing the performance of conventional convolutional networks. The RepViTBlock (RVB) combines Depthwise Convolution and Channel Interaction mechanisms to achieve significant performance improvements. The RVB structure is shown in Fig 3. The RepViT block optimizes feature extraction through depthwise convolution and channel interaction. In contrast to the original RepViT block, this version incorporates the SE module for attention. The main path of this architecture integrates 3 × 3 depthwise convolution (DW) [24] and channel feature interaction into a unified branch configuration during the inference stage. In addition, 1 × 1 Extended Convolutional layers (EConv) [25] and 1 × 1 projection layers (Player) [26] are used for inter-channel interaction, significantly enhancing the diversity and expressiveness of features. Compared to the main path, the interaction path reduces the component of channel feature interaction. The combination of outputs from both branches forms the final output of the RVB. The BMR-YOLO model incorporates an optimized attention mechanism, improving its capacity to capture spatial and contextual data across multiple scales. This enhancement enables the model to perform exceptionally well in feature learning for fall detection, particularly in challenging environments like low-light and partial occlusion. Specifically, the RVB consists of the following key formulas:

(1) Depthwise Convolution (DW): Depthwise convolution (DW convolution) significantly improves the efficiency of neural networks by reducing computational load and the number of parameters. In traditional convolution, each input channel is convolved with every output channel, resulting in a high computational cost. In contrast, depthwise convolution uses one filter per input channel, which greatly decreases both the computational demand and the parameter count. Moreover, depthwise convolution helps reduce the model size, which in turn enhances operational efficiency on devices with limited computational resources, particularly in low-power environments such as mobile and embedded devices. The formula for DW is as follows:

$$Y_{h,w,c}=\sum_{i=0}^{K-1}\sum_{j=0}^{K-1}X_{h+i,w+j,c}\cdot K_{i,j,0,c},$$ (1)

Where: (*Y*_*h*,*w*,*c*_ is the value at position *h*, *w* in the output tensor, at channel *c*; *X*_*h* + *i*,*w* + *j*,*c*_ is the corresponding value at the same position in the input tensor; and *K*_*i*,*j*,0,*c*_ represents the weight of the convolution kernel at position *i*, *j* for channel *c*.

(2) Squeeze-and-Excitation Module (SE): The SE module enhances the network’s focus on important features by adaptively weighting the features of each channel, thereby improving the model’s representational power. It is particularly effective in complex tasks, as it captures contextual information more effectively. At the same time, it reduces redundancy and optimizes the selective expression of features without significantly increasing computational cost, leading to an overall improvement in network performance. The formulas for Squeeze and Excitation are shown in Eqs (2) and (3), respectively.

$$z_{c}=\frac{1}{HW}\sum_{i=1}^{H}\sum_{j=1}^{W}x_{x,j,c},$$ (2)

$$s_{c}=\sigma \left({𝐖}_{2}\left({𝐖}_{1}z_{c}\right)\right),$$ (3)

In the squeeze step (*X*_*i*,*j*,*c*_), represents the value at position (*i*, *j*) and channel *c* in the input feature map, while *Z*_*c*_ is the global feature of channel *c*. In the excitation step, *sigma* refers to the Sigmoid activation function, which ensures that the weights of each channel stay within the range of [0, 1]. *W*_1_ and *W*_2_ are the weight matrices of the fully connected layers, and represents the channel features obtained from the Squeeze step.

(3) Extended Convolutional (Econv): Econv is a variant of Convolutional Neural Networks (CNNs) commonly used to enhance the receptive field of convolution operations or to process specific input data more efficiently. Extended convolution modifies the convolution operation by expanding the kernel or increasing the stride, allowing the network to capture a broader range of contextual information. The formula for Econv is shown below:

$$y(t)=\sum_{i=0}^{N-1}X(t+ri)w(i),$$ (4)

Here, *x*(*t*) represents the value of the input signal at time *t*; *w*(*i*) is the value of the convolutional kernel at position *i*; r is the expansion factor (usually an integer greater than 1); when r > 1, the spacing between the elements of the convolutional kernel is increased; and N is the length of the convolutional kernel.

(4) Projection Layers (Player): In neural networks, particularly in deep learning, the player function serves to map data from one space to another. It is commonly used to change the dimensions of the data, perform feature transformation, or achieve specific tasks such as dimensionality reduction, encoding, and more. The formula for the player is shown below:

y=Wx+b, (5)

Here, *x* is the input vector; *W* is the weight matrix, responsible for performing the projection, mapping the input to the new space; b is the bias term, also aiding in the mapping to the new space; and y is the output after the projection.

#### 3.2.3 MultiSEAMhead.

**Fig 4:**
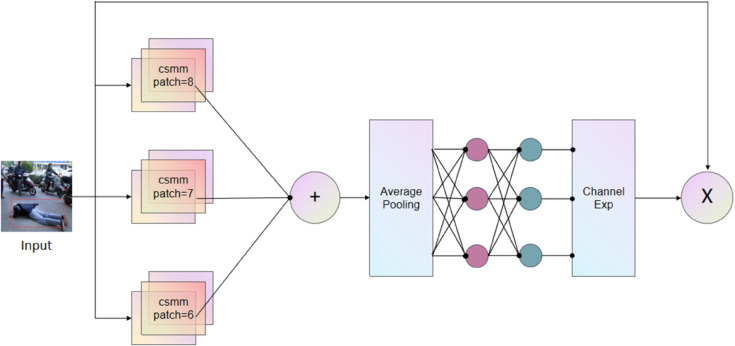
Architecture of the MultiSEAM module. Integrated into the detection head, this mechanism tackles occlusion by leveraging depthwise separable convolutions, pointwise convolution for feature fusion, and a channel expansion module to integrate multi-scale context, thereby enhancing focus on informative features.

In crowded public spaces or places with physical obstacles, fall detection is often affected, making detection more challenging. The MultiSEAM attention mechanism proposed by Ziping Yu et al. effectively addresses the detection problem in occluded scenarios. This paper incorporates MultiSEAM attention-assisted detection into the YOLOv8 detection head. The attention mechanism is centered around depthwise separable convolution (CSMM) and residual connections, optimizing both computational efficiency and model stability for complex tasks. Although depthwise separable convolutions effectively reduce parameters and enhance the learning of individual channel significance, they are constrained in their capacity to model interactions between different channels. In order to tackle this, the outputs from convolutions at different depths are fused via a pointwise (1x1) convolution. Subsequently, the CSMM module’s output features are processed with an average pooling module to integrate multi-level features, enhancing the model’s ability to capture information at various scales. In the end, the Channel Exp module efficiently combines multi-scale features, resulting in improved performance in image recognition. The attention mechanism’s structure is presented in Fig 4.

#### 3.2.4 Improvement of loss function.

In object localization tasks, the choice of loss function plays a critical role in determining the positioning accuracy of detected regions, which ultimately governs the system’s recognition capability. The Intersection over Union (IoU) measure serves as a fundamental evaluation criterion for assessing alignment precision between estimated regions and their corresponding annotated areas. This versatile measurement approach proves particularly valuable for quantifying output quality in tasks involving spatial region estimation. Specifically, the IoU computation determines spatial congruence by analyzing the ratio between overlapping and combined areas of predicted and reference regions.

However, under challenging conditions such as partial obstructions, scale variations, and inter-target overlaps commonly encountered in fall detection scenarios, conventional CIoU loss demonstrates limited effectiveness. To address this, we propose substituting the baseline CIoU with the more advanced SIoU (SCYLLA IoU) formulation. Experimental evidence suggests that in crowded public settings, the SIoU loss demonstrates superior adaptability and consistent performance, effectively enhancing model training efficiency while reducing convergence time.

**Fig 5:**
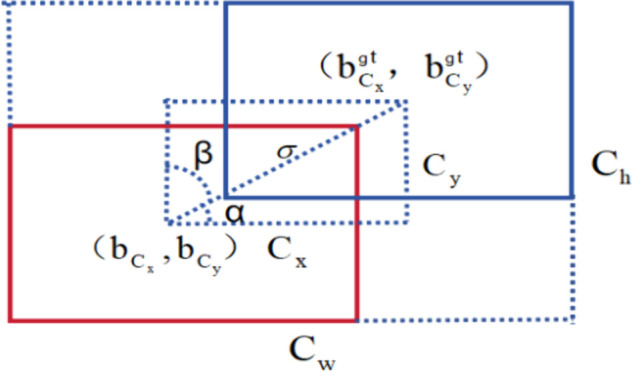
Components of the SIoU loss function. SIoU introduces direction-aware regression by combining Angle Cost (Λ), Distance Cost (*Δ*) weighted by angle, Shape Cost (Ω), and traditional IoU Cost (*L*_*IoU*_). This guides the model to a more efficient convergence path for superior localization accuracy.

The SIoU metric incorporates directional alignment considerations between estimated and ground-truth regions when calculating localization accuracy, introducing enhanced geometric constraints into the regression optimization process. The four key components of the revised loss function will be introduced in the following equationsThe revised loss function is shown in Fig 5, the blue rectangle denotes the true bounding box, while the red rectangle represents the predicted one. Among them, $\left(b_{C_{x}}^{gt},b_{C_{y}}^{gt}\right)$ is the center coordinate of the real box, and $\left(b_{C_{x}},b_{C_{y}}\right)$ is the center coordinate of the predicted box; The horizontal extent (*C*_*w*_) and vertical span (*C*_*h*_) correspond to the measurements of the minimally enclosing rectangular region that contains both the reference annotation and the detector’s output region; the distance, denoted as *σ*, is calculated between the midpoints of both the predicted and real bounding boxes; the width difference (*C*_*x*_) and height difference (*C*_*y*_) refer to the differences between the midpoints of the two bounding boxes, respectively. The calculation process of the four functions is represented by the following formula.

(1) Angle loss: The smallest angle is formed by the line joining the centers of the two boxes and the x-y plane, where Λ=0 when the center point is aligned on the x-axis or y-axis. When the line connecting the center point forms a 45^°^ angle with the x-axis, Λ=1. The definition of angle loss is provided in Formula (1).

$$\Lambda =1-2sin^{2}[sin^{-1}(sin\alpha )-\frac{\Pi }{4}],$$ (6)

(2) Distance loss: It indicates the separation between the central points of the two objects, with its penalty cost increasing in proportion to the angle cost. The definition of distance loss is provided in Formula (2).

$$\Delta =\sum_{t=x,y}(1-e^{-\gamma \rho_{t}})=2-e^{-\gamma \rho_{x}}-e^{-\gamma \rho_{y}},$$ (7)

Among them γ=2−Λ, $\rho_{x}=(\frac{b_{C_{x}}^{gt}\;-\;b_{C_{x}}}{C_{w}}{)}^{2}$, $\rho_{y}=(\frac{b_{C_{y}}^{gt}\;-\;b_{C_{y}}}{C_{w}}{)}^{2}$. Because of the angle loss effect, the impact of distance loss is relatively minimal when the predicted and true boxes are horizontally aligned. As the angle between the center points approaches 45^°^, the contribution of distance loss becomes more significant.

(3) Shape loss: the aspect ratio between two boxes is defined by measuring the difference in either length or width and comparing it to the maximum ratio of length or width between the two. The shape loss is mathematically defined in Formula (3).

$$\Omega =(1-e^{-\omega_{w}}{)}^{\Theta }+(1-e^{-\omega_{h}}{)}^{\Theta },$$ (8)

Here, $\omega_{w}$ is the proportion of the absolute width variation between the predicted and true boxes relative to the maximum value, while $\omega_{h}$ indicates the proportion of the absolute height variation relative to the maximum value; Θ is the weight coefficient for shape loss.

(4) IoU loss: the SIoU loss function’s final expression is provided in Formula (4).

$$L_{SIoU}=1-L_{IoU}+\frac{\Delta +\Omega }{2},$$ (9)

## 4 Experiment and analysis

### 4.1 Dataset

#### 4.1.1 Data sources.

High-quality and diverse datasets are crucial for the effective learning and good generalization ability of models, reducing the risk of overfitting and ensuring stable and reliable performance in real-world applications. Therefore, selecting appropriate datasets and performing sufficient preprocessing and labeling are the foundations for improving model performance. To ensure that the datasets are representative and diverse, this study uses two datasets: one is a self-labeled public setting dataset, and the other is the publicly available UR Fall Detection (URFD) dataset. These two datasets cover a wide range of scenes and conditions, enhancing the model’s adaptability under various circumstances.

#### 4.1.2 Data collection process.

Considering that existing public datasets often lack challenging scenarios such as occlusion and low-light conditions, we have constructed a specialized dataset focusing on fall events in these complex environments. This custom dataset, named the BMR-fall dataset, is designed to assess and enhance the robustness of models in real-world challenging conditions. It contains 11,000 images from real fall events, extracted from video footage and meticulously annotated. Each image was labeled using the LabelMe tool, with bounding boxes drawn around individuals exhibiting fall behaviors to indicate the fall event.

In terms of environmental distribution, the BMR-fall dataset includes approximately 70% of images featuring occlusion in crowds, and 30% of images involving low-light environments, ensuring comprehensive coverage of challenging real-world scenarios. Additionally, the URFD dataset contains 70 different activity sequences, 30 of which are fall events, and 40 are daily activities. These data were captured using ground and ceiling cameras to provide multi-angle views. This multi-perspective and multi-person setup enhances the model’s ability to accurately detect falls in various environments, including situations with multiple individuals.

**Fig 6:**
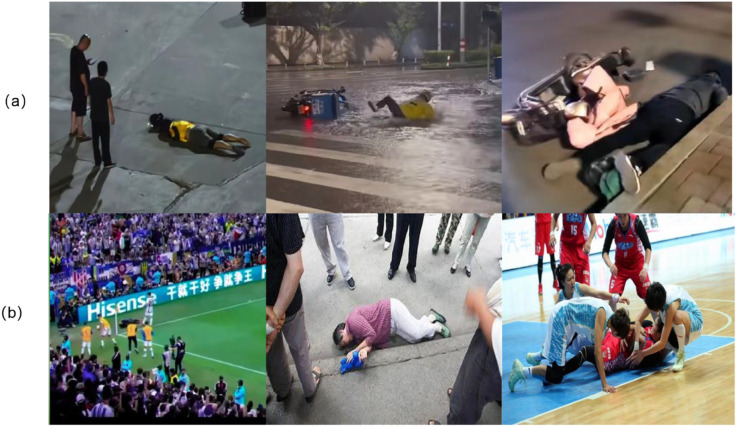
Example images from the BMR-Fall dataset. The dataset provides benchmarks for challenging real-world conditions: (a) low-light conditions with degraded image quality, and (b) occlusion scenarios where the target is obscured by people or objects, ensuring robust model validation.

As shown in Fig 6, the collection process of the BMR dataset is illustrated. The proposed BMR-fall dataset specifically emphasizes fall events occurring under complex environmental conditions, such as occlusion and low-light scenarios, thus filling a critical gap in existing fall detection data resources.

Regarding ethical considerations, all images used in this study are sourced from publicly available data and do not contain any personally identifiable information or sensitive content. As the data is publicly accessible and appropriately anonymized, ethical committee approval is not required for this research. Access to all datasets adheres to the respective platform’s terms of service, and privacy protection measures were thoroughly applied throughout the data processing procedure. In addition, the publicly available datasets used in this study do not contain any personally identifiable information, and therefore, institutional review board (IRB) approval is not required.

### 4.2 Evaluation indicators

To assess the viability of the experimental design, this study employs several performance indicators, each defined mathematically below. The first metric quantifies the proportion of true positive predictions relative to all positive classifications, reflecting the model’s prediction correctness. Meanwhile, the second metric measures the fraction of actual positive cases correctly recognized by the model, demonstrating its detection capability for target instances. The computational methods for these indicators are expressed as follows:

$$P=\frac{TP}{TP+FP},$$ (10)

$$R=\frac{TP}{TP+FN},$$ (11)

In the formula, FN represents a false counterexample; TP represents true proportion; FP stands for false positive example. AP quantifies the region under the Precision-Recall curve for every individual class, and the classifier’s quality is determined by the area size. The calculation formula is as follows:

$$AP=\int_{0}^{1}PdR,$$ (12)

MAP is calculated as the average of AP values over several categories. The calculation formula is as follows:

$$mAP=\frac{\sum_{i=1}^{k}AP_{i}}{k},$$ (13)

MAP can be subdivided into two metrics: mAP@0.5 and mAP@0.5:0.95. When the threshold is set at 50%, mAP@0.5 indicates the model’s average precision. On the other hand, mAP @ 0.5: 0.95 is obtained by averaging the mAP scores over the threshold range from 50% to 95%. This approach offers a thorough and precise assessment of the model’s performance across varying thresholds, showcasing its ability to adapt flexibly to different precision demands.

FLOPs are widely employed to assess a model’s computational complexity and its need for hardware processing power. Under the same hardware conditions, smaller GFLOPs have higher computational efficiency and are easier to deploy.

### 4.3 Results and analysis

#### 4.3.1 Algorithm comparison results.

1. Parameter Settings
  The experimental conditions used in this study are presented in Table 1, with the system featuring an Nvidia GeForce RTX 2080Ti GPU. To ensure sufficient model convergence, the experiment is set up with an input image resolution of 640 pixels, a training mini-batch of 16, and a total of 300 iterations.
2. Statistical tests
  To rigorously validate the effectiveness of performance improvement, we conducted a statistical significance analysis. All the data listed in Table 2 are based on the mean ± standard deviation (mean ± std) from five independent training and evaluation processes using different random seeds. In the analysis, we used a paired t-test to compare the mAP@0.5 of BMR-YOLO with other models. The extremely low p-values (all less than 0.001) indicate that the performance improvement of BMR-YOLO is statistically significant, and this enhancement cannot be attributed to random fluctuations alone. Additionally, we calculated the 95% confidence interval (CI) of the performance improvement relative to the primary benchmark model, YOLOv8n, which ranged from [3.8%, 5.6%]. This result provides a reliable estimate of the expected performance gain. This analysis further demonstrates the robustness and reliability of the proposed improvements.
3. Algorithm Comparison
  To verify the advantages of the improved BMR-YOLO model in fall detection algorithms, Table 3 shows a performance comparison of the original YOLOv8 algorithm, current mainstream algorithms, and the improved YOLOv8 algorithm. To guarantee the experiment’s fairness and reliability, all algorithms will be evaluated under the same testing conditions, guaranteeing the fairness and accuracy of the comparison results.

**Table 1:** Hardware and software environment configuration for the experiment in this article.

Schedule	Capacity
Operating System	ubuntu18.04
CPU	Intel Core i7-9700K(3.60GHz)
CPU	Nvidia GeForce RTX 2080 Ti
Memory	15GB
Python	python-3.8
CUDA	CUDA 11.4
PyTorch Frame	torch-2.1.1

**Table 2:** The results are reported as mean ± standard deviation (mean ± std) from five independent runs. The p-values from the paired t-test and the 95% confidence interval (CI) for the performance differences (*Δ*mAP) between BMR-YOLO and each comparative model are shown in the table.

Model	mAP@0.5 (Mean ± Std)	vs. BMR-YOLO (p-value)	Improvement (ΔmAP)	95% CI of ΔmAP
BMR-YOLO	0.899 ± 0.004	-	-	-
YOLOv8n	0.852 ± 0.005	< 0.001	+0.047	[0.038, 0.056]
YOLOv9t	0.864 ± 0.006	< 0.001	+0.035	[0.028, 0.042]
YOLOv10n	0.857 ± 0.005	<0.001	+0.042	[0.035, 0.049]
YOLOv5s	0.855 ± 0.007	<0.001	+0.044	[0.035, 0.053]
YOLOv7-tiny	0.819 ± 0.008	<0.001	+0.080	[0.069, 0.091]
YOLOv3-tiny	0.724 ± 0.010	< 0.001	+0.175	[0.162, 0.188]

**Table 3:** Algorithm comparison test results.

Model	Precision	Recall	mAP@0.5	mAP@0.5:0.95	GFLOPs
YOLOv3-tiny	0.720	0.721	0.724	0.369	19.0
YOLOv5s	0.806	0.831	0.855	0.525	15.8
YOLOv7-tiny	0.737	0.841	0.819	0.405	13.2
YOLOv8n	0.822	0.831	0.852	0.451	8.7
YOLOv9t	0.837	0.821	0.864	0.474	7.6
YOLOv10n	0.836	0.793	0.857	0.452	6.5
BMR-YOLO	0.877	0.838	0.899	0.557	6.5

Experimental results demonstrate that the BMR-YOLO framework attains higher detection precision than alternative approaches, recall, average accuracy, and computational power in fall detection. Regarding detection precision (mAP@0.5), the proposed method exhibits a 7.16% mean enhancement relative to comparative approaches; in terms of the computational power GFLOPs of the model, Comparative analysis demonstrates a mean reduction of 4.3 relative to alternative approaches, making it more convenient to deploy under the same hardware conditions. Although the computational power of YOLOv9t model is consistent with that of BMR-YOLO model, BMR-YOLO’s mAP@0.5 compared to YOLOv9t [27], it has improved by 3.5% and has excellent performance in model detection accuracy. The calculation ability of the YOLOv10n model is 1.0 less than that of the BMR-YOLO model, however, the BMR-YOLO model’s performance in terms of mAP@0.5 has dropped by 4.2%, and the accuracy and recall rate are inferior to that of the BMR-YOLO model. The reason why GFLOPs of BMR-YOLO model increased by 1.0 compared with YOLOv10n is that the inclusion of the BiFormer attention mechanism in the backbone enhances the model’s computational power while significantly boosting its detection capability. In general, BMR-YOLO outperforms YOLOv10n [28] in terms of overall performance. The experiment has demonstrated that the improved model BMR-YOLO has certain advantages over the original model and other models in terms of comprehensive evaluation of accuracy and computational power.

Several tests were performed to assess the effect of incorporating the BiFormer mechanism into the BMR-YOLO model, comparing it with four different attention mechanisms, SimAM [29], Triple [30], MPCA [31], and MLCA [32], which have simpler structures and improved detection accuracy. As evidenced in Table 4, the BiFormer attention module yields substantial performance gains over four competing methods under identical test settings, while the increase in computational complexity is minimal. These results indicate that the BiFormer architecture achieves an optimal trade-off between model accuracy and resource utilization, establishing it as the superior choice.

**Table 4:** Comparison of ablation experiments.

Model	Precision	Recall	mAP@0.5	mAP@0.5:0.95	GFLOPs
SimAM	0.817	0.816	0.846	0.447	8.1
Triplet	0.847	0.807	0.857	0.469	8.1
MPCA	0.818	0.828	0.863	0.478	8.1
MLCA	0.810	0.842	0.855	0.462	8.1
BiFormer	0.844	0.853	0.879	0.541	8.3

#### 4.3.2 Ablation experiment.

To assess the performance of the model, we conducted ablation experiments using a specially designed fall detection dataset. Detailed experimental results can be found in Table 5. In the table, blank cells indicate that the respective approach was applied to enhance the model, while “×” indicates that the corresponding method was not used by the model.

**Table 5:** Comparison of ablation experiments.

BiFormer	MultiSEAM-head	C2f_rvb	SIoU	Precision	Recall	mAP@0.5	mAP@0.5:0.95	GFLOPs
×	×	×	×	0.822	0.831	0.852	0.451	8.7
	×	×	×	0.844	0.853	0.879	0.541	8.3
		×	×	0.861	0.805	0.8888	0.549	8.1
			×	0.856	0.825	0.890	0.552	6.5
				0.877	0.838	0.899	0.557	6.5

Table 5 highlights that the optimized YOLOv8n model achieves considerable advancements in multiple performance metrics. First, after incorporating the BiFormer attention mechanism module, the model’s mAP@0.5 increased from 0.845 to 0.89, and the mAP@0.5 0.95 rose from 0.451 to 0.552. At the same time, the GFLOPs reduced from 8.7 to 8.3, this led to improvements in the model’s precision while simultaneously reducing its computational demands, emphasizing the crucial contribution of the BiFormer module to enhancing model performance.

Next, when the MultiSEAMhead detection head was introduced for optimization, the mAP@0.5 further improved to 0.888, and the mAP@0.5:0.95 increased to 0.541, while the GFLOPs dropped to 7.3. This demonstrates the superior capability of the detection head in enhancing accuracy and reducing computational complexity. With this improvement, the model’s computational efficiency was significantly increased, and its performance became even more outstanding.

The most notable optimization was achieved by simultaneously applying the BiFormer attention mechanism module, the MultiSEAMhead detection head, and the SIoU loss function. In this combined optimization approach, mAP@0.5 improved by 5.4%, mAP@0.5:0.95 increased by 10.6%, and GFLOPs were reduced by 1.2. These results demonstrate that the integrated approach enhances prediction precision while simultaneously decreasing processing requirements. Through this complete set of optimizations, BMR-YOLO achieved the best performance across multiple dimensions, fully showcasing the deep optimization effects of our experiments.

In conclusion, after these key improvements, BMR-YOLO showed significant gains in accuracy, recall, mean precision, and computational efficiency, achieving optimal overall performance. The experimental results clearly demonstrate that these carefully designed optimizations not only enhanced the model’s accuracy and efficiency but also delivered outstanding performance in practical applications, thoroughly proving the effectiveness of our improvement strategy.

#### 4.3.3 Experimental results.

**Fig 7:**
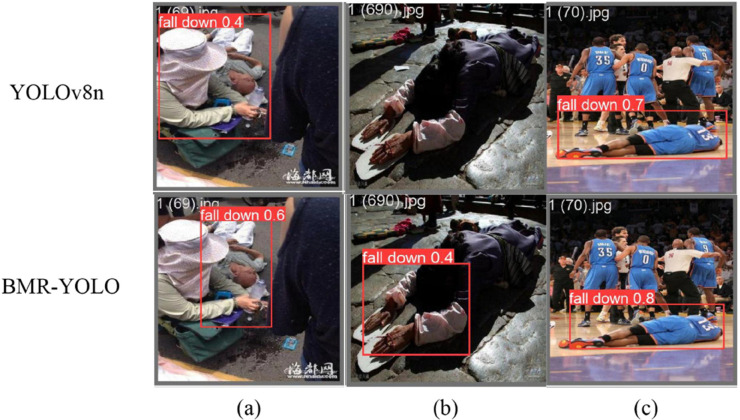
Detection result comparison: YOLOv8nvsBMR-YOLO. Qualitative results demonstrate BMR-YOLO’s superiority: (a) it reduces missed detections (false negatives); (b) it minimizes false alarms (false positives); (c) it provides more precise bounding boxes, confirming enhanced reliability in complex environments.

Based on the experimental results mentioned above, we successfully optimized the BMR-YOLO model and compared it with the previous version of the YOLOv8n model. Fig 7(a) and 7(b) reveal that the YOLOv8n framework encounters false negatives and false positives when tested in complex environments, such as crowd occlusion or low-light conditions, leading to unstable performance in practical applications. In contrast, the optimized BMR-YOLO model remains stable and accurate in detecting fall events, even in these challenging scenarios, demonstrating its superiority in complex environments.

Additionally, as depicted in Fig 7(c), the BMR-YOLO model surpasses the YOLOv8n framework, even in more straightforward scenarios, providing more precise detection results. These results confirm that BMR-YOLO achieves superior detection capability in basic environments while maintaining exceptional adaptability and computational effectiveness in complex scenarios. Therefore, whether in more complex environments or simpler ones, the BMR-YOLO model consistently outperforms the YOLOv8n model, making it a more adaptable and effective solution, especially in the context of the current research.

#### 4.3.4 Model performance.

**Fig 8:**
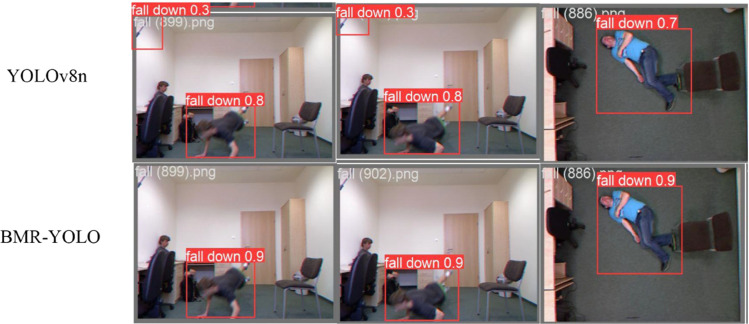
Performance comparison between YOLOv8n and BMR-YOLO models on the UR Fall dataset based on benchmarking results.

**Fig 9:**
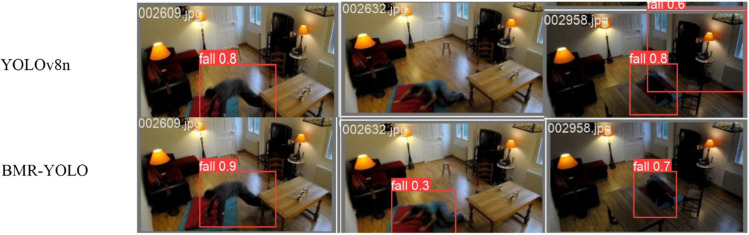
Generalization performance of the BMR-YOLO model on the Lei2 Fall dataset based on generalization validation.

1. Benchmark Tests
  Comprehensive benchmarking was conducted using the publicly available UR Fall Detection Dataset. As shown in Table 6, BMR-YOLO demonstrates superior detection capability compared to YOLOv8n, with notable improvements across multiple evaluation criteria: classification accuracy (Precision), detection sensitivity (Recall), mean average precision at both 0.5 and 0.5:0.95 IoU thresholds, and computational efficiency (GFLOPs). These results indicate that BMR-YOLO not only excels in detection accuracy but also demonstrates stronger generalization capabilities, this enhances its versatility, enabling application across a diverse array of scenarios. As shown in Fig 8, the YOLOv8n model exhibits noticeable missed detections when processing this dataset, whereas BMR-YOLO reliably and accurately detects fall events. Comparative analysis reveals that BMR-YOLO achieves high detection accuracy on both datasets, especially in complex environments. BMR-YOLO’s robust generalization ability establishes it as a leader in the field of fall detection, this further validates its capacity to handle more complex situations and yield higher accuracy in detection results.
2. Generalization Capability
  To evaluate the generalization ability of the BMR-YOLO model, we conducted a generalization test on the Le2i Fall dataset. As shown in Table 7, BMR-YOLO significantly outperforms YOLOv8n across several key metrics, including classification precision, recall, mAP at both 0.5 and 0.5:0.95 IoU thresholds, and computational efficiency (GFLOPs). These results demonstrate that BMR-YOLO not only excels in detection accuracy but also exhibits outstanding cross-scenario generalization ability, further enhancing its flexibility in real-world applications. As shown in Fig 9, YOLOv8n experiences noticeable missed detections on this dataset, while BMR-YOLO achieves more reliable and accurate fall event detection. A comprehensive comparative analysis indicates that BMR-YOLO maintains high detection precision across different datasets, especially in complex environments. Its robust generalization capability highlights the model’s leadership in the field of fall detection, providing strong evidence of its ability to handle complex scenarios and improve detection accuracy.

**Table 6:** Benchmark results on the UR Fall dataset.

Model	Precision	Recall	mAP@0.5	mAP@0.5	GFLOPs
YOLOv8n	0.893	0.85	0.917	0.619	8.7
BMR-YOLO	0.954	0.842	0.935	0.629	6.5

**Table 7:** Generalization Validation on the Lei2 Fall Dataset.

Model	Precision	Recall	mAP@0.5	mAP@0.5	GFLOPs
YOLOv8n	0.867	0.713	0.821	0.386	8.7
BMR-YOLO	0.88	0.84	0.894	0.456	6.5

## 5 Conclusions

This paper presents an innovative improvement to the YOLOv8n algorithm—BMR-YOLO, aimed at addressing the fall detection problem in complex scenarios, especially under challenging conditions such as crowd occlusion. Through extensive experimental validation, BMR-YOLO has demonstrated excellent detection performance across various environments, particularly excelling in improving accuracy and generalization ability. Compared to traditional algorithms, BMR-YOLO significantly enhances the reliability of fall detection in public spaces, effectively dealing with complexities like occlusion, and provides strong support for the successful deployment of fall detection technology on terminal devices.

Although BMR-YOLO has achieved significant results in complex scenarios, future research can continue to focus on innovations in lightweight optimization techniques. By optimizing the model size and enhancing inference efficiency, we can significantly improve the deployment efficiency and user experience of mobile devices and embedded systems, while maintaining exceptional performance. This advancement contributes significantly to achieving accurate and timely fall detection in practical applications, effectively ensuring personal safety. We are confident in the future of the BMR-YOLO model and look forward to its ability to adapt and perform even better in more complex and dynamic scenarios. As this technology continues to evolve, we believe it will be widely applied in real-time monitoring systems and smart security management, bringing greater convenience and security to people’s lives.

## Supporting information

S1 Data (ZIP)
