# Supplementary figures and images for: BMR-YOLO: A deep learning approach for fall detection in complex environments
Source: PLoS One. 2025 Nov 7;20(11):e0335992. doi: 10.1371/journal.pone.0335992 (PMC12594333; doi:10.1371/journal.pone.0335992)

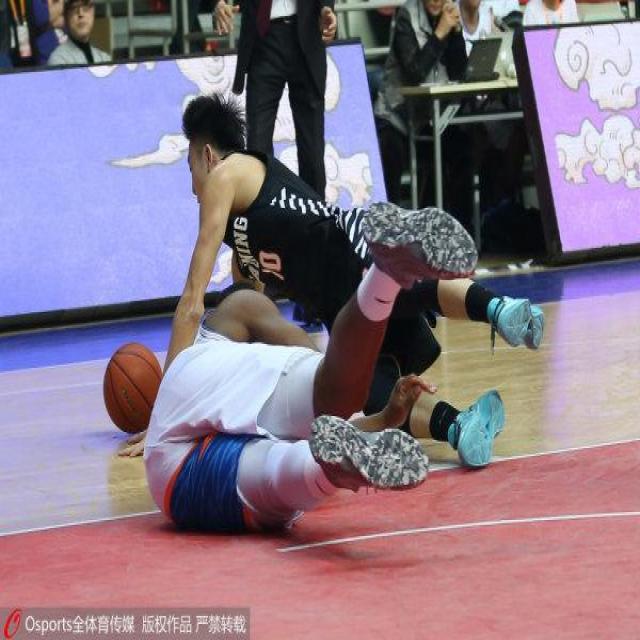

Supplement: S1 Data — (ZIP) [file pone.0335992.s001.zip › S1_Data/images/1.jpg]

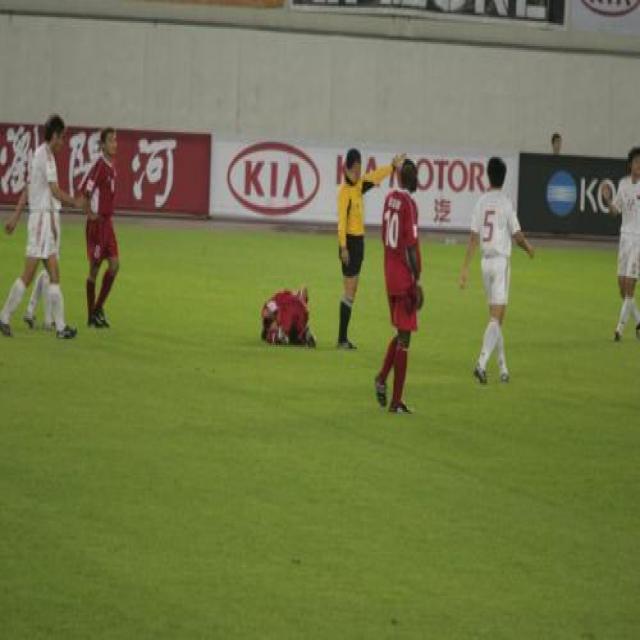

Supplement: S1 Data — (ZIP) [file pone.0335992.s001.zip › S1_Data/images/10.jpg]

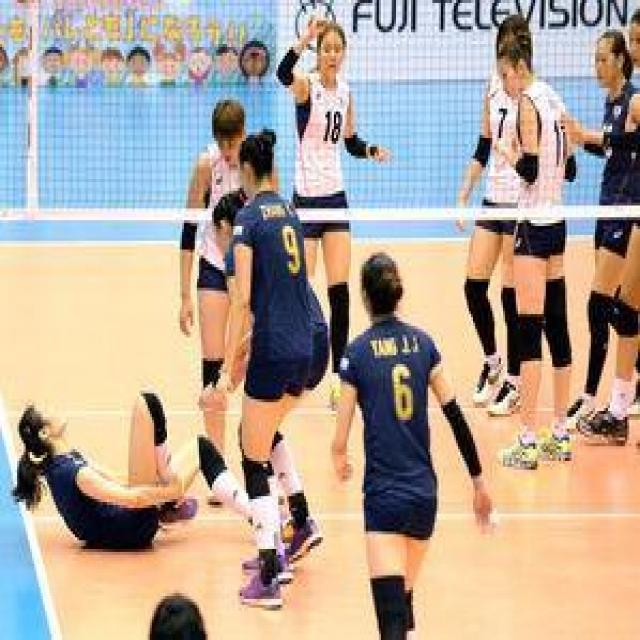

Supplement: S1 Data — (ZIP) [file pone.0335992.s001.zip › S1_Data/images/11.jpg]

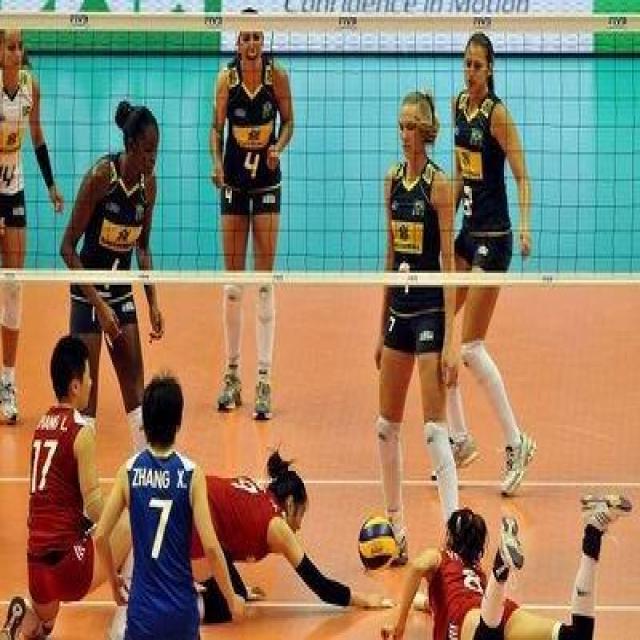

Supplement: S1 Data — (ZIP) [file pone.0335992.s001.zip › S1_Data/images/12.jpg]

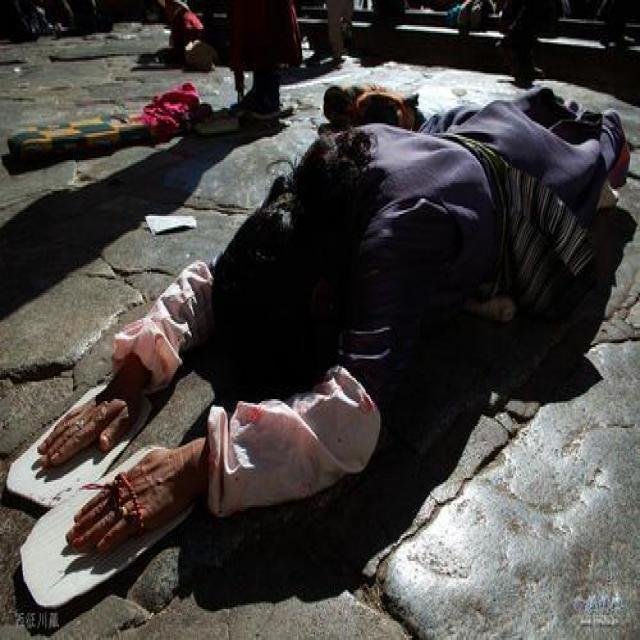

Supplement: S1 Data — (ZIP) [file pone.0335992.s001.zip › S1_Data/images/13.jpg]

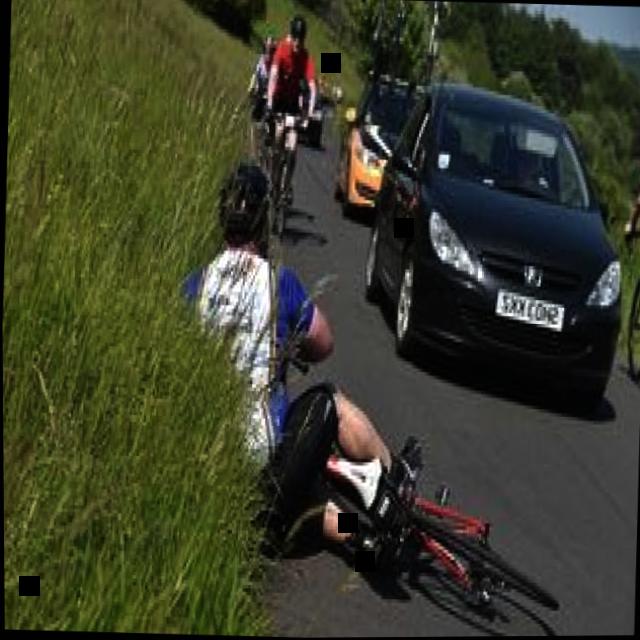

Supplement: S1 Data — (ZIP) [file pone.0335992.s001.zip › S1_Data/images/14.jpg]

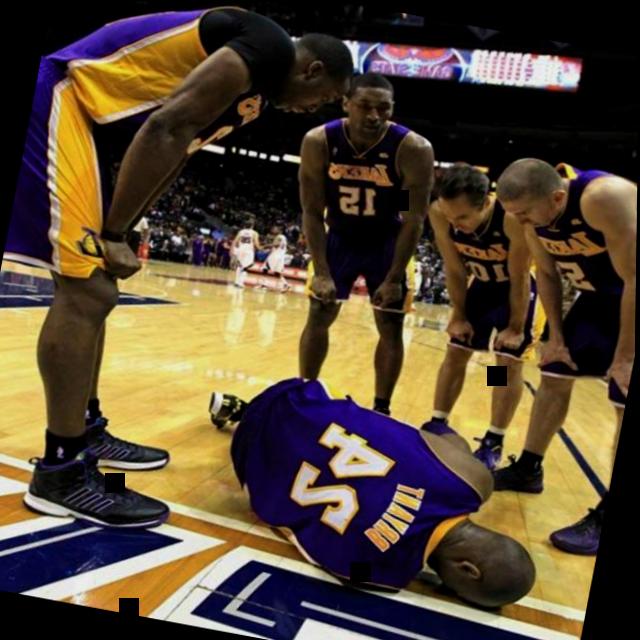

Supplement: S1 Data — (ZIP) [file pone.0335992.s001.zip › S1_Data/images/15.jpg]

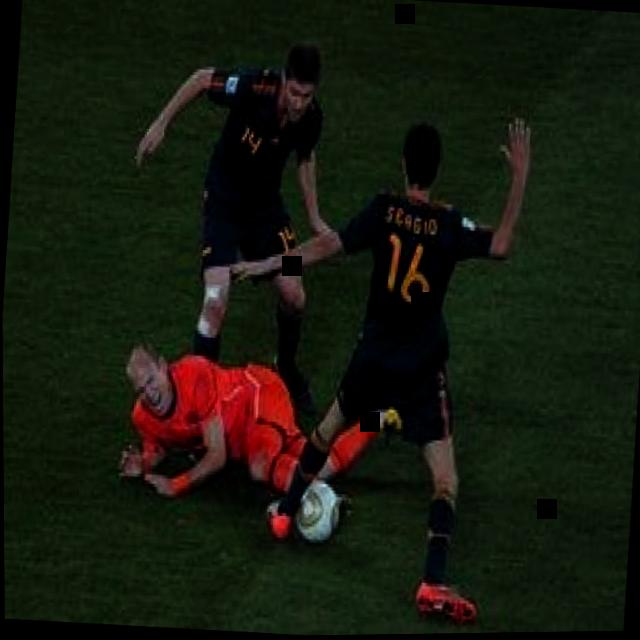

Supplement: S1 Data — (ZIP) [file pone.0335992.s001.zip › S1_Data/images/16.jpg]

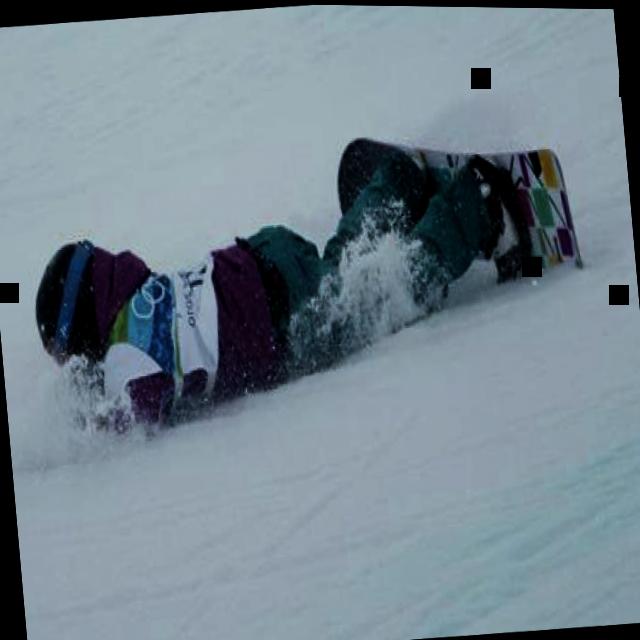

Supplement: S1 Data — (ZIP) [file pone.0335992.s001.zip › S1_Data/images/17.jpg]

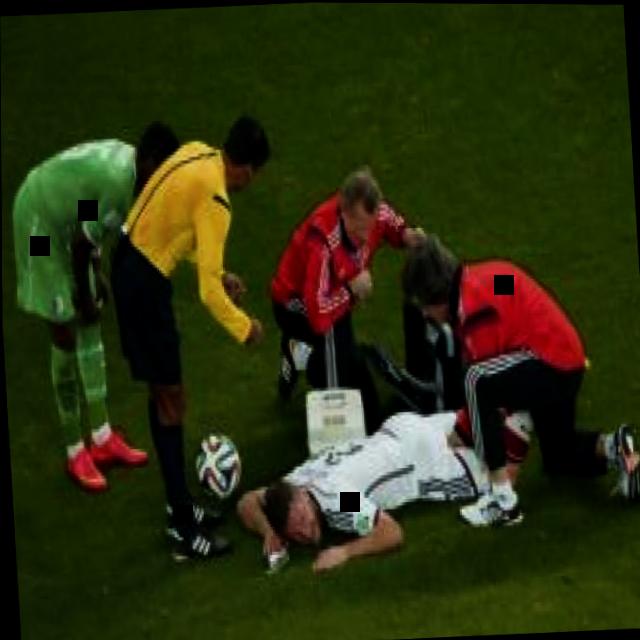

Supplement: S1 Data — (ZIP) [file pone.0335992.s001.zip › S1_Data/images/18.jpg]

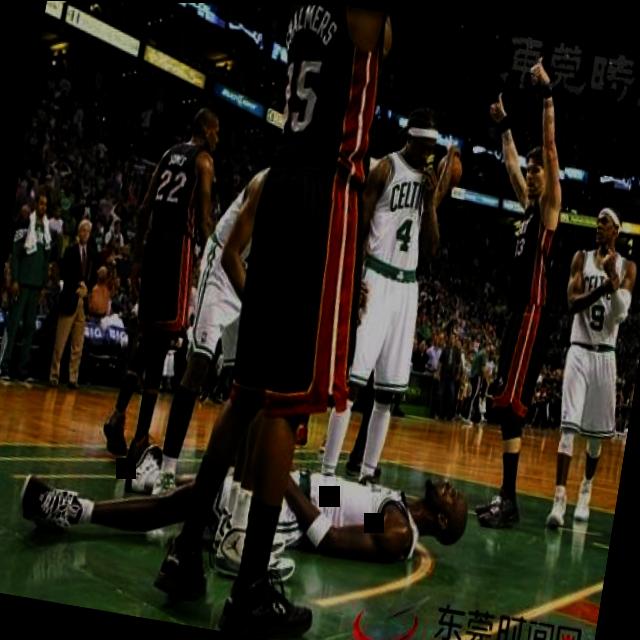

Supplement: S1 Data — (ZIP) [file pone.0335992.s001.zip › S1_Data/images/19.jpg]

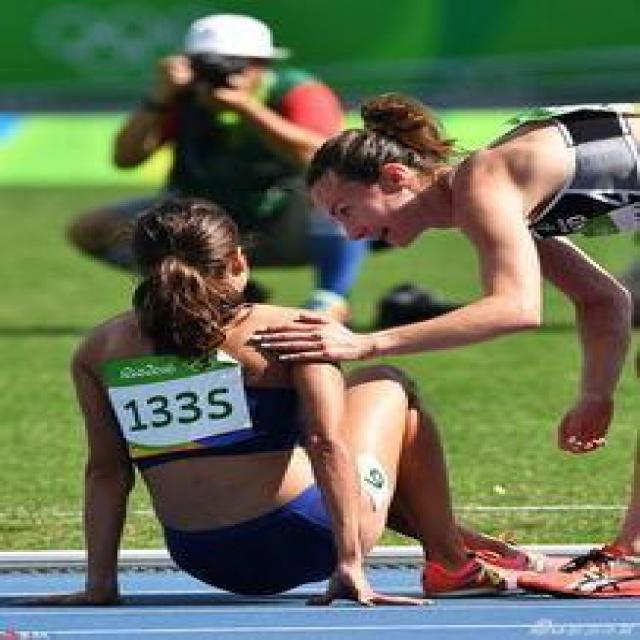

Supplement: S1 Data — (ZIP) [file pone.0335992.s001.zip › S1_Data/images/2.jpg]

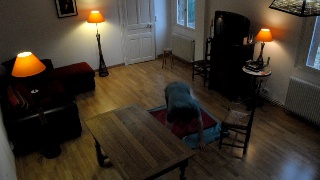

Supplement: S1 Data — (ZIP) [file pone.0335992.s001.zip › S1_Data/images/20.jpg]

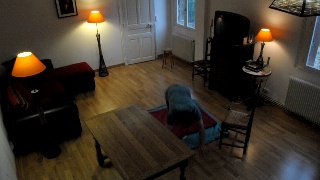

Supplement: S1 Data — (ZIP) [file pone.0335992.s001.zip › S1_Data/images/21.jpg]

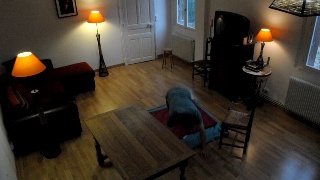

Supplement: S1 Data — (ZIP) [file pone.0335992.s001.zip › S1_Data/images/22.jpg]

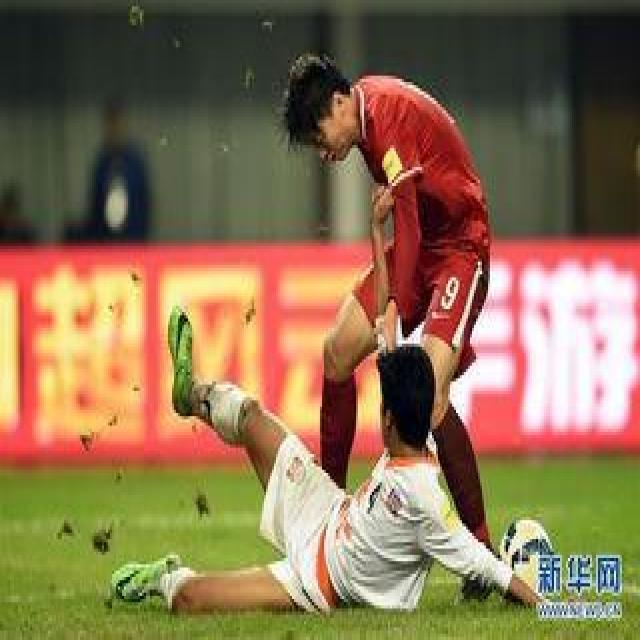

Supplement: S1 Data — (ZIP) [file pone.0335992.s001.zip › S1_Data/images/3.jpg]

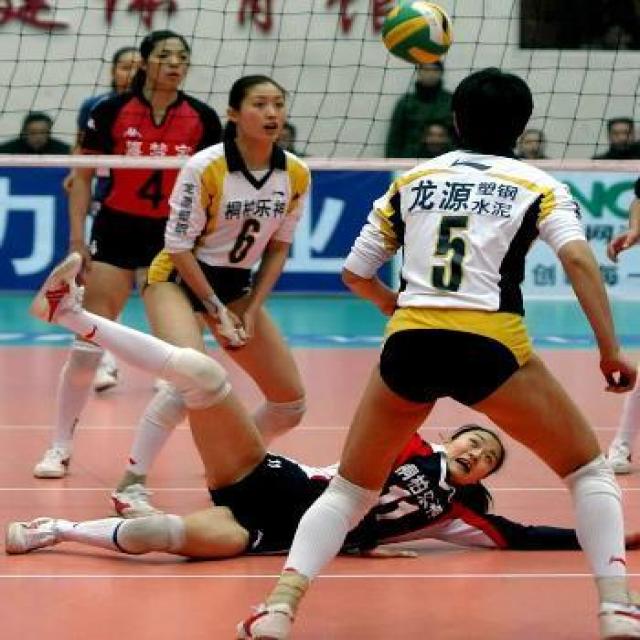

Supplement: S1 Data — (ZIP) [file pone.0335992.s001.zip › S1_Data/images/4.jpg]

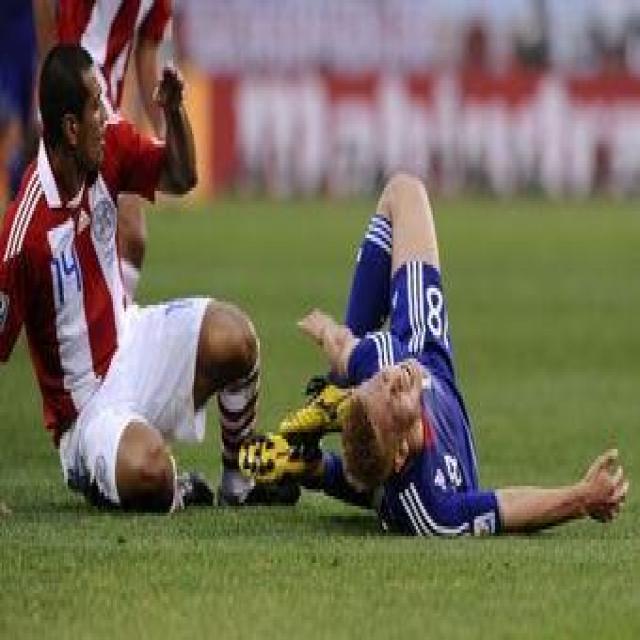

Supplement: S1 Data — (ZIP) [file pone.0335992.s001.zip › S1_Data/images/5.jpg]

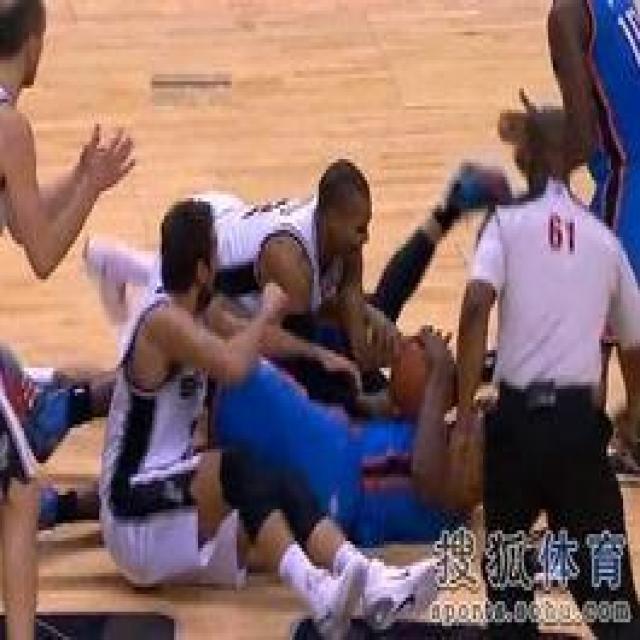

Supplement: S1 Data — (ZIP) [file pone.0335992.s001.zip › S1_Data/images/6.jpg]

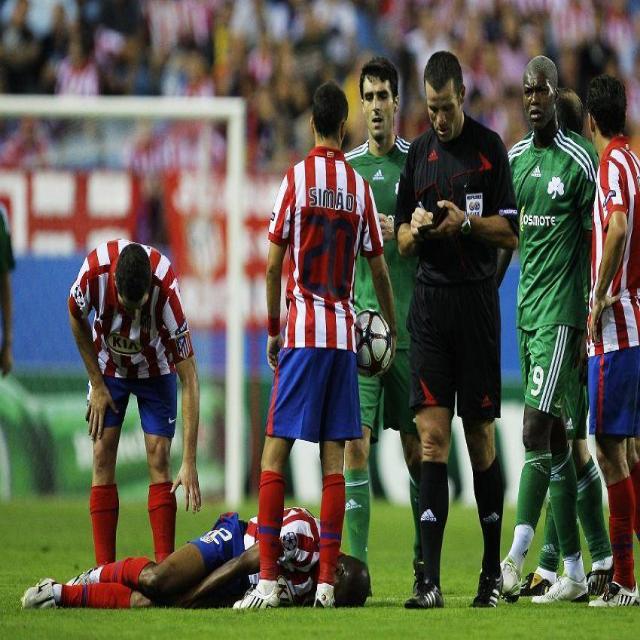

Supplement: S1 Data — (ZIP) [file pone.0335992.s001.zip › S1_Data/images/7.jpg]

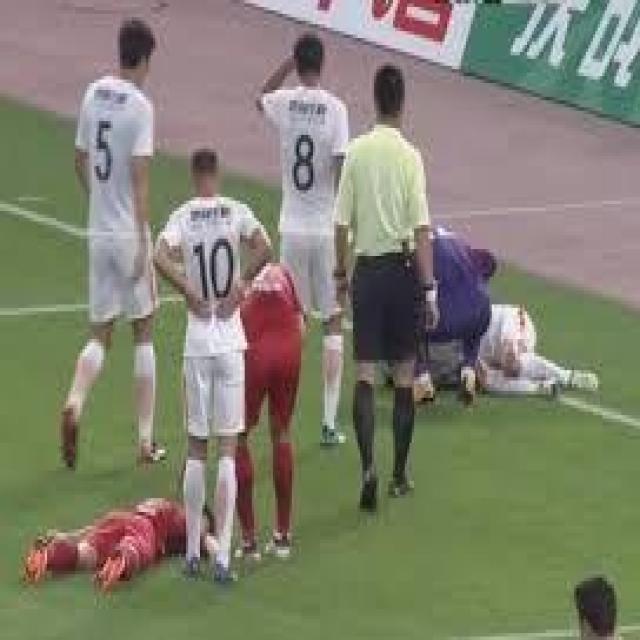

Supplement: S1 Data — (ZIP) [file pone.0335992.s001.zip › S1_Data/images/8.jpg]

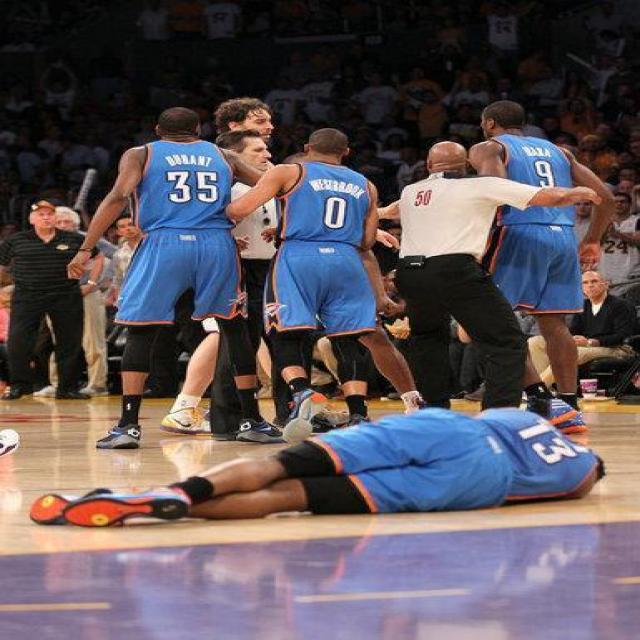

Supplement: S1 Data — (ZIP) [file pone.0335992.s001.zip › S1_Data/images/9.jpg]
